# Estimation of Lens Stray Light with Regard to the Incapacitation of Imaging Sensors—Part 2: Validation

**DOI:** 10.3390/s22239447

**Published:** 2022-12-02

**Authors:** Bastian Schwarz, Gunnar Ritt, Bernd Eberle

**Affiliations:** Fraunhofer IOSB, Gutleuthausstr. 1, 76275 Ettlingen, Germany

**Keywords:** stray light, imaging sensor, scatter parameters, Harvey scatter model, laser safety, FRED

## Abstract

Recently, we developed a simple theoretical model for the estimation of the irradiance distribution at the focal plane of commercial off-the-shelf (COTS) camera lenses in case of laser illumination. The purpose of such a model is to predict the incapacitation of imaging sensors when irradiated by laser light. The model is based on closed-form equations that comprise mainly standard parameters of the laser dazzle scenario and those of the main devices involved (laser source, camera lens and imaging sensor). However, the model also includes three non-standard parameters, which describe the scattering of light within the camera lens. In previous work, we have performed measurements to derive these typically unknown scatter parameters for a collection of camera lenses of the Double-Gauss type. In this publication, we compare calculations based on our theoretical model and the measured scatter parameters with the outcome of stray light simulations performed with the optical design software FRED in order to validate the reliability of our theoretical model and of the derived scatter parameters.

## 1. Introduction

When an imaging sensor system (e.g., a surveillance camera or a thermal imager) is illuminated with laser radiation of appropriate wavelength and power/pulse energy, the sensor system may become incapacitated [1,2,3,4]. Incapacitation means that the sensor system can no longer fulfill its intended task, either through reversible (non-damaging) effects, such as dazzle, or through irreversible effects because of sensor damage. Whether incapacitation occurs or not depends on the spatial light distribution at the focal plane of the imaging sensor system. The light distribution can appear quite complex due to scattering of the laser light at the optical and mechanical parts of the camera lens and is rarely comparable to a simple Airy diffraction pattern as described in textbooks, neither in shape nor in size.

Unlike in the civilian sector where the occurrence of dazzle or detector damage to cameras from laser radiation can be disruptive, in military operations, laser irradiation poses a real threat [5,6,7,8] since safety aspects are concerned. Thus, it would be of advantage to be able to perform laser protection calculations for cameras, following the well-established laser safety concepts that exist for the human eye. The established laser safety quantities related to the human eye are the maximum permissible exposure (MPE) at the cornea of the eye and, in connection with the MPE, the nominal ocular hazard distance (NOHD), e.g., see [9,10]. Corresponding quantities related to the reversible effects of laser dazzle of the human eye are the maximum dazzle exposure (MDE) and nominal ocular dazzle distance (NODD) [11,12,13].

In earlier work [14], we defined similar quantities for imaging sensors:

- Maximum permissible exposure of a sensor, MPE_S_: The maximum applicable laser irradiance at the entrance aperture of the camera lens to prevent the sensor from being damaged.
- Nominal sensor hazard distance, NSeHD: The hazard distance corresponding to the MPE_S_.
- Maximum dazzle exposure of a sensor, MDE_S_: Laser irradiance at the entrance aperture of the camera lens that corresponds to a certain dazzle level (for definition, see below).
- Nominal sensor dazzle distance, NSeDD: The hazard distance corresponding to the MDE_S_.

**Figure 1:**
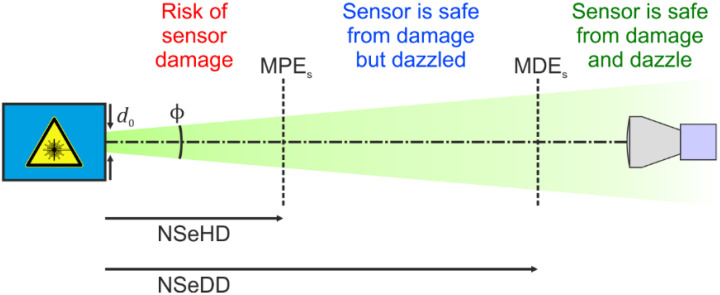
Exposure limits for sensors (maximum permissible exposure for a sensor, MPE_S_, and maximum dazzle exposure for a sensor, MDE_S_) and corresponding hazard distances (nominal sensor hazard distance, NSeHD, and nominal sensor dazzle distance, NSeDD). Taken from reference [14].

An illustration of these quantities is shown in Figure 1.

The aim of our earlier work was also to establish equations with which the quantities defined above could be calculated under the following constraints:

- Objective 1. Equivalent to laser safety calculations for the human eye, the values of MPE_S_ and MDE_S_ shall be stated at the position of the entrance aperture of the camera lens.
- Objective 2. The equations for the laser safety quantities shall be given as closed-form expressions containing only well-known operations and functions. The equations should be as simple as possible but still sufficiently accurate.
- Objective 3. The equations for the laser safety quantities should incorporate, as far as practical, only standard parameters of the involved devices (laser, camera lens, and imaging sensor) as specified by the manufacturer and, taking into account the underlying scenario (e.g., distance and atmospheric extinction).

The formulated objectives have the following background: Objective 1 allows the user to position a power meter at the typically easily accessible place in front of the camera lens in order to compare the calculated exposure values with the incident laser irradiance. Objective 2 ensures that users who do not have relevant experience in this field can still perform laser protection calculations for sensors. In principle, everybody should be able to perform laser safety calculations using a sheet of paper and a pocket calculator. Therefore, we want to avoid numerical calculations that can only be performed with the help of a computer. Finally, Objective 3 shall enable the user to perform such calculations for a wide range of sensor systems, i.e., for different combinations of camera lenses and camera sensors, without the necessity to measure unknown parameters beforehand.

To establish laser safety quantities for sensors analogous to the human eye (exposure limit MPE and hazard distance NOHD), we had to start from the damage threshold of the sensor, which is located at the focal plane of the camera lens. Such laser-induced damage thresholds (LIDT) for imaging sensors are normally not known and have to be determined by appropriate measurements, e.g., see the work of Schwarz et al. [15,16,17]. In a further step, we had to transfer the sensor’s damage threshold to a corresponding value at the position of the camera lens’ entrance aperture in order to achieve Objective 1. However, this requires finding out how the irradiance distribution of the threatening laser beam is related to the irradiance distribution in the focal plane of the camera lens. This issue has been the biggest difficulty in our efforts to define the laser safety quantities for sensors since there is not, as with the human eye, only one single kind of camera lens but many different ones. The irradiance distribution at the focal plane depends on the design and quality of the camera lens, comprising its scattering characteristics and image distortions. As a rule, the scattering properties of camera lenses are usually only very rarely known and must otherwise be determined in time-consuming measurements with a dedicated setup.

The same considerations are also valid for laser dazzle quantities. For example, the laser dazzle threshold can be defined as the irradiance, where the pixels of the imaging sensor start to saturate. Such saturation thresholds can be calculated easily from the specifications of the imaging sensor [14]. In order to describe the extent of laser dazzle, such as the size of the dazzle spot on the imaging sensor, we again need the quantitative irradiance distribution at the focal plane.

In summary, this means the following: the basis for achieving our goal was to set up a theoretical model based on closed-form equations that quantitatively describe the irradiance distribution at the focal plane of a camera lens in the case of laser irradiation. With some minor compromises, we were able to achieve the objectives formulated above:

- Regarding Objective 2, we had to apply several simplifications and approximations in order to obtain easily manageable closed-form equations for a non-experienced user. However, this means that in this way, calculated laser safety quantities are just estimates and may differ for other sensor systems.
- Regarding Objective 3, threshold values for sensor saturation $E_{\mathrm{sat}}$ and laser-induced sensor damage $E_{\mathrm{dam}}$ must be known to perform laser safety calculations. These threshold values are typically non-standard parameters. While estimates for the saturation threshold $E_{\mathrm{sat}}$ of a sensor can be derived from the sensor specifications, this is usually not possible for laser damage thresholds $E_{\mathrm{dam}}$. However, such values may be found in the literature, e.g., [15,16,17,18,19].

Furthermore, it is known from the literature that the scattering of light at the lens has an important influence on the spatial distribution of the laser light at the imaging sensor [20,21]. Thus, our theoretical model required to include the scattering characteristics of the camera lens. To account for light scattering from the smooth surfaces of the optical elements of the camera lens, we used three scatter parameters in our equations. These scatter parameters do not belong to the standard specifications and are therefore usually not known for COTS camera lenses. Therefore, we performed dedicated measurements to estimate such scatter parameters for a selection of eight typical COTS camera lenses of the Double-Gauss type [22]. Based on these measurements, we derived a generic set of scatter parameters that can be used when the manufacturer does not specify such parameters. Of course, there are deviations from this generic set with respect to real camera lenses.

More detailed considerations on this shall be postponed to Section 3, after we have introduced our theoretical model and the experimental derivation of the scattering parameters.

In this publication, we present our efforts to verify whether the scattering parameters for camera lenses derived in our previous work [22] are reliable, as well as the validation of our theoretical model of reference [14]. For this, we first measured the stray light parameters of a two-element laser focusing optics consisting of a well-known optical layout. Subsequently, we performed stray light simulations for this optics using the optical engineering software FRED [23]. If our theoretical model and our experimentally determined scattering parameters are reliable, then the irradiance distributions obtained with the FRED software on the one hand and with the calculations of our theoretical model on the other hand should agree when using our measured scattering parameters.

In addition to the tests regarding the laser-focusing optics, we performed stray light simulations for a generic camera lens of the Double-Gauss type, whose optical layout was extracted from a standard textbook on optical design. To do so, we applied our above-mentioned generic set of scatter parameters derived from our earlier work [22]. Thus, we compared the output of our theoretical model to the FRED simulation results in order to validate the applicability of our model in conjunction with our generic set of scatter parameters regarding an arbitrary lens of the Double-Gauss type.

In Section 2, we present a review of our earlier work. Then, at the end of Section 2, we lay the basis to understand better the compromises mentioned above, which will be the topic of Section 3. In Section 4, we report on our experimental work related to the determination of the scatter parameters of a two-element laser focusing optics, performed especially for this publication. Section 5 is dedicated to our simulation work, where we present our approach and compare the output of the simulation with the corresponding calculations of our theoretical model.

## 2. Basics

In Section 2.1, we first review the basics of our simple theoretical model to estimate the irradiance distribution at the focal plane of a camera lens. Then, in Section 2.2, we summarize our measurements and the corresponding data analysis to derive the essential scatter parameters for camera lenses, which are a fundamental part of the input to the theoretical model. Both topics will be discussed only very briefly and thus, for a more in-depth description, we refer the reader to our previous work [14,22]. The content of Section 2.1 and Section 2.2 was taken from references [14,22] and is presented below in a summarized form.

### 2.1. Theoretical Model

**Figure 2:**
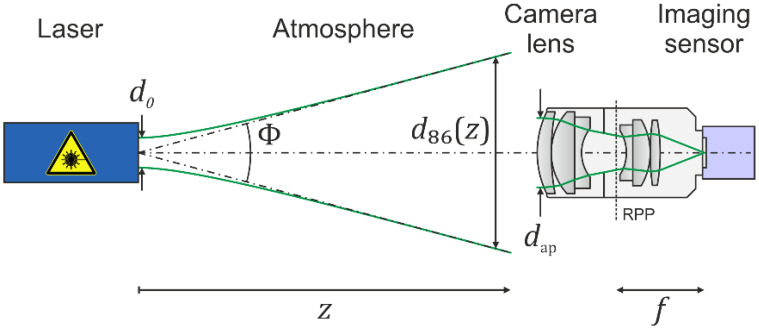
Schematic view of our dazzle scenario. RPP: rear principal plane. Please note that the location and size of the apertures and pupils of the camera lens are drawn for illustrative purposes only. Based on Figure 2 of reference [14].

The theoretical model of our earlier publication [14] assumes a scenario as depicted in Figure 2. A laser emits a beam with Gaussian beam shape, characterized by output power $P_{0}$, wavelength λ, output diameter $d_{0}$ and full angle divergence Φ. The laser beam illuminates a sensor system consisting of a camera lens and an imaging sensor. The laser beam diameter at the camera lens is denoted by $d_{86}$ or $d_{63}$, depending on whether the irradiance refers to 1/e² or 1/e points of the peak irradiance, respectively. These two quantities are related by

(1) $$d_{86}=\sqrt{2}\cdot d_{63}$$

The camera lens is described by the focal length f and the diameter of the entrance pupil $d_{\mathrm{ap}}$. Further parameters of a camera lens are the f-number $F=f/d_{\mathrm{ap}}$, the number of optical elements $N_{\mathrm{oe}}$, the transmittance T and the scatter parameters s, $b_{0}$ and l.

The ratio of the beam diameter $d_{86}$ to the diameter of the camera lens’ entrance pupil $d_{ap}$ is called truncation factor ν, which has a determining influence on the distribution of the laser light in the focal plane of the camera lens:

(2) $$\nu =d_{86}/d_{\mathrm{ap}}$$

Taking into account the truncation factor ν, the laser power entering the lens can be calculated as follows:

(3) $$P_{\mathrm{in}}=P_{0}\;\left(1-\exp\left(-\frac{2}{\nu^{2}}\right)\right)$$

In our theoretical model, the incident power contributes to the focal plane irradiance distribution $E_{\mathrm{fp}}$ by two components: (a) the scatter/stray light component $E_{s}$ and (b) the diffraction component $E_{d}$:

(4) $$E_{\mathrm{fp}}\left(r\right)=E_{s}\left(r\right)+\eta_{d}\;E_{d}\left(r\right),$$

where $\eta_{d}$ describes the fraction of the incident laser power that is diffracted and r is the radial coordinate. In Equation (4), as well as in all subsequent equations, the dependency on the radial coordinate r (in the focal plane) can be replaced by the dependency on the viewing angle Θ using the relationship

(5) $$\Theta =\frac{r}{f}\;\Leftrightarrow \;r=\Theta f.$$

#### 2.1.1. Scatter Component

To estimate the contribution of the stray light $E_{s}$ to the focal plane irradiance distribution, we rely on the work of G. L. Peterson, who published an analytical approach for this task [24]. According to Peterson’s work, the stray light irradiance $E_{s}$ at the focal plane of an optical system with $N_{\mathrm{ss}}$ scattering surfaces is simply calculated as the sum of the contributions from the single scattering surfaces:

(6) $$E_{s}\left(r\right)=\overset{N_{\mathrm{ss}}}{\underset{j=1}{\sum }}E_{s,j}\left(r\right)$$

The contribution of the *j*th scattering surface $E_{s,j}$ is given by

(7) $$E_{s,j}\left(r\right)=\pi T\;NA^{2}\frac{a_{\mathrm{ent}}^{2}}{a_{j}^{2}}b_{0}{\left[1+{\left(\frac{NA\;r}{la_{j}}\right)}^{2}\right]}^{\frac{s}{2}}E_{\mathrm{ent}},$$

where T is the optic’s transmittance, NA the numerical aperture, $a_{\mathrm{ent}}$ the radius of the beam at the first scattering element, $a_{j}$ the radius of the beam at the *j*th scattering element and $E_{\mathrm{ent}}$ the incident irradiance. Equation (7) is identical to Equation (20) of reference [24], which was derived by Peterson using the three-parameter Harvey scatter model as a bidirectional scattering distribution function (BSDF). This type of BSDF describes the angular distribution of stray light caused by scatter from the smooth surfaces of optical elements using the three parameters s, $b_{0}$ and l. Other sources of stray light, such as multiple reflections or scatter from the lens housing, are not considered in Equation (7). For a detailed explanation of the BSDF and the meaning of the scatter parameters, we refer the reader to other publications, e.g., reference [25,26].

For our theoretical model, we applied some simplifications to Equations (6) and (7) to keep them manageable for a typical camera lens with five or more optical elements $N_{\mathrm{oe}}\geq 5$. First, we transformed the summation in Equation (6) to a simple multiplication with the factor $N_{\mathrm{ss}}$ by defining the beam diameter at each scattering surface $a_{j}=\mathrm{const}.=a_{\mathrm{ent}}$ to be constant. Second, we further assumed that the number of scattering surfaces is twice the number of optical elements: $N_{\mathrm{ss}}=2\cdot N_{\mathrm{oe}}$, since we typically do not know how many optical elements are cemented together in a COTS camera lens.

These two simplifications finally lead to the reduced equation

(8) $$E_{s}\left(r\right)=\frac{P_{\mathrm{in}}TN_{\mathrm{ss}}b_{0}}{f^{2}}\frac{1}{{\left(v^{*}\right)}^{2}}{\left[1+\frac{1}{{\left(v^{*}\right)}^{2}}\cdot {\left(\frac{r}{lf}\right)}^{2}\right]}^{\frac{s}{2}},$$

where $v^{*}$ is defined by

(9) $$\nu^{*}=\min\left(1,\frac{\nu }{\sqrt{2}}\right).$$

This modified truncation factor $\nu^{*}$ takes into account that the beam diameter within the camera lens will not increase, if a laser beam, which is already much larger than the entrance aperture, expands further, e.g., with increasing distance to the laser source.

The ratio of scattered power to the incident power (for a single scattering surface) is called the total integrated scatter (TIS) and can be calculated by [27]

(10) $$TIS=\{\begin{array}{r} 2\pi b\frac{100^{s}}{s+2}\;\left[{\left(1+l^{2}\right)}^{\frac{s+2}{2}}-{\left(l^{2}\right)}^{\frac{s+2}{2}}\right],\;s\neq -2\\ 2\pi b\frac{{\left(100l\right)}^{s}}{2}l^{2}\ln\left(1+\frac{1}{l^{2}}\right),\;s=-2 \end{array}$$

with the alternative scatter parameter *b*, which is related to the already introduced quantity $b_{0}$ by

(11) $$b_{0}=b\cdot {\left(100l\right)}^{s}.$$

The quantity TIS is used to calculate the fraction $\eta_{d}$ of the incident power that is diffracted; see Equation (4).

#### 2.1.2. Diffraction Component

Our theoretical model assumes a Gaussian beam. Thus, for our calculations, we apply the diffraction pattern of a truncated Gaussian beam, e.g., see reference [28]. This diffraction pattern in the focal plane of the sensor can be thought of as something between an Airy diffraction pattern and a pure Gaussian distribution. The shape depends on the value of the truncation factor *v* and consists of a central lobe that can be approximated by a Gaussian distribution and diffraction rings of a lower power, similar to the Airy diffraction pattern.

The central lobe can be approximated by

(12) $$E_{\mathrm{GA}}\left(r\right)=E_{0}\left(\nu \right)\exp\left(-8\frac{r^{2}}{d_{\mathrm{spot}}^{2}}\right)$$

where the peak irradiance of the diffraction pattern is given by

(13) $$E_{0}\left(\nu \right)=\frac{P_{\mathrm{in}}T\pi }{4\lambda^{2}F^{2}}\cdot \frac{2\nu^{2}{\left[1-\exp\left(-\frac{1}{\nu^{2}}\right)\right]}^{2}}{1-\exp\left(-\frac{2}{\nu^{2}}\right)}$$

and the spatial extent of the central lobe is calculated by [28]

(14) $$d_{\mathrm{spot}}=k\lambda F.$$

Here, *k* is a spot size constant, which also depends on the truncation factor ν see reference [28] for details. However, for a typical camera lens designed for the visible spectral range, the diffraction spot size $d_{\mathrm{spot}}$ is usually in the order of some micrometers. Since the pixel size of common CCD or CMOS imaging sensors is of the same order, the central lobe is usually not resolved by an imaging sensor.

Outside the central lobe, the wings of the diffraction pattern can be approximated by the mean of the diffraction ring irradiance, which is given by

(15) $$E_{\mathrm{dr}}\left(r\right)=\frac{P_{\mathrm{in}}T\lambda F}{\pi^{3}r^{3}}\cdot \frac{\frac{2}{\nu^{2}}\exp\left(-\frac{2}{\nu^{2}}\right)}{1-\exp\left(-\frac{2}{\nu^{2}}\right)}.$$

Finally, the radial irradiance distribution $E_{d}\left(r\right)$ in the focal plane due to diffraction can be calculated by

(16) $$E_{d}\left(r\right)=\{\begin{array}{c} E_{\mathrm{cl}}\left(r\right),\;\left|r\right|\leq r_{\mathrm{cl}}\\ E_{\mathrm{dr}}\left(r\right),\;\left|r\right|>r_{\mathrm{cl}} \end{array},$$

where $r_{\mathrm{cl}}$ is the radial coordinate, which separates the central lobe of the diffraction pattern from the diffraction rings.

Using the total integrated scatter TIS of Equation (10), the fraction of power that is diffracted by the sensor’s lens can be calculated as

(17) $$\eta_{d}={\left(1-TIS\right)}^{N_{\mathrm{ss}}}$$

Equation (17) contains the number of scattering surfaces $N_{\mathrm{ss}}$, since the quantity TIS is defined for a single scattering surface only.

### 2.2. Experimental Determination of Scatter Parameters

In our theoretical model, the scatter component $E_{s}$ of the focal plane irradiance distribution depends on the scatter parameters s, $b_{0}$ and l. Unfortunately, these scatter parameters are usually not known for COTS camera lenses. In our earlier work, we thus performed a series of measurements on a selection of eight typical camera lenses with focal lengths ranging from 25 mm to 100 mm [22]. We derived the radial irradiance profiles for these camera lenses when illuminated with laser radiation and subsequently fitted our model equations to these irradiance profiles, by using the quantities s, $b_{0}$ and l as fit parameters. The outcome of that work was a set of scatter parameters for each of the tested camera lenses. Here, we give a short review of the experimental setup, the data acquisition process and the data analysis procedure to derive the relevant scatter parameters of camera lenses. For details, we refer the reader to reference [22].

#### 2.2.1. Experimental Setup

**Figure 3:**
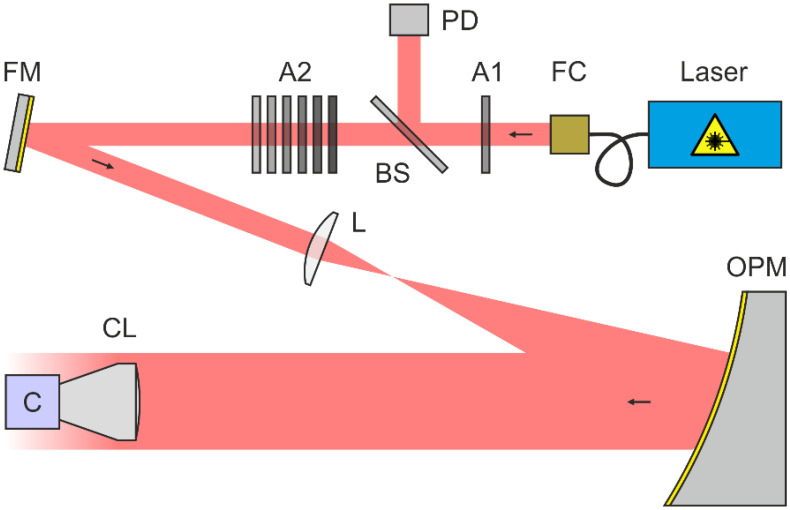
Scheme of the experimental setup for the measurement of the irradiance distribution in the focal plane of camera lenses. FC: fiber collimator, A1/A2: attenuator, BS: beam splitter, PD: reference photodiode, FM: folding mirror, L: focusing lens, OPM: off-axis parabolic mirror, CL: camera lens, C: camera. Taken from reference [22].

A scheme of our experimental setup to measure the irradiance distribution at the focal plane of a camera lens is shown in Figure 3. All relevant details can be found in [22].

As a light source, we used a multi-wavelength laser device that offered four different laser wavelengths of 488 nm, 515 nm, 561 nm and 640 nm, each with an output power of several tens of milliwatts. Using a fiber collimator, FC, the laser light was collimated. A first attenuator A1 limited the maximum laser power to a value in the order of 2 µW. Subsequently, the reflected light of a beam splitter BS was sent to a calibrated reference photodiode PD. The transmitted light passed a second attenuator A2, consisting of a set of neutral density filters. In the further course, the laser beam passed a folding mirror FM and was then expanded by a Keplerian telescope with magnification M=6.7 consisting of a focusing lens L and an off-axis parabolic mirror OPM. Finally, the collimated laser beam was sent to the camera lens CL under test. Depending on the laser wavelength, the laser beam diameter at the entrance of the camera lens was around 20.5 mm. In order to measure the spatial irradiance distribution at the focal plane of the camera lens, we used camera C as a detector.

#### 2.2.2. Data Acquisition

The main challenge in measuring the irradiance distribution of a focused laser beam is the high dynamic range of irradiance values, which has to be covered. The irradiance at the center of our laser spot is quite high (~10^4^ W/m^2^), whereas the off-center stray light irradiance is quite low (~10^−4^ W/m^2^). Typically, the dynamic range of a camera device is in the order of 60 dB, but we need to cover eight orders of magnitude. Thus, a single image will only deliver a part of the complete radial irradiance profile with a linear signal response. In order to obtain the desired irradiance information for almost the complete area of the imaging sensor, eight images had to be acquired using different combinations of exposure time $t_{\exp}$ and laser power $P_{0}$. The exposure times for such a measurement ranged from 100 µs up to 100 ms; the laser power was controlled using neutral density filters A2 with optical densities ranging from OD=4 to OD=0 (no filter in the beam path). Measurements were performed for each of the four available laser wavelengths and were repeated for different f-numbers *F* of the camera lens in order to obtain a sufficiently large data set for a camera lens.

#### 2.2.3. Data Analysis

Since the amount of recorded image data is typically quite huge, we used automated analysis software to derive the scatter parameters. Briefly, this included the following steps:

**Step 1: Radial profile generation**

**Figure 4:**
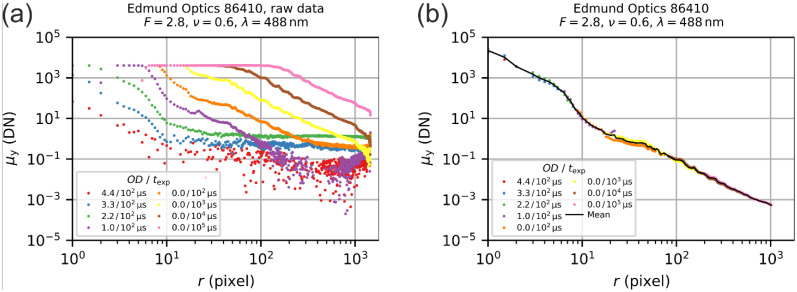
Example of the data analysis: (**a**) Result of the irradiance profile generation, (**b**) final result after averaging the partly overlapping single profiles. Based on Figures 11 and 12 of reference [22].

From the image data, a radial irradiance profile was assessed for each acquired image by averaging the pixel values for each occurring radial distance r to the center of the laser spot. As an example, Figure 4a shows the result of the radial profile generation process for the camera lens Edmund Optics 86410. These data were taken from our previous work [22].

**Step 2: Profile stitching**

Due to the limited dynamic range of a camera, it was necessary to merge all eight radial profiles of a measurement to get a complete radial profile for the specific parameters used (camera lens under test, f-number F and laser wavelength λ). This was carried out by the following:

1. Filtering out overexposed and underexposed values from the derived profiles.
2. Subsequent normalization of each profile according to the individual camera exposure time, the laser power used for the image acquisition and the optical density of attenuator A2.
3. Averaging of the scaled profiles; see Figure 4b.

**Step 3: Curve fitting**

Fit of the theoretical curve according to Equation (4) to the radial irradiance profiles of a measurement series (comprising the measurements with all four laser wavelengths) using the scatter parameters s, b and l as fit parameters.

For this, the irradiance values of the theoretical model were converted to grey values $\mu_{y}$ of the camera according to the EMVA 1288 standard [29] by using the equation

(18) $$\mu_{y}=K\cdot \eta \cdot \frac{EAt_{\exp}}{\frac{hc}{\lambda }}+\mu_{y.\mathrm{dark}}$$

where E is the irradiance at the pixel, A is the pixel area, $t_{\exp}$ is the camera’s exposure time, h is the Planck constant, c is the vacuum speed of light, λ is the laser wavelength, η is the quantum efficiency, K is the overall system gain, and $\mu_{y.\mathrm{dark}}$ is the dark signal. K and η for the camera used were determined in advance; see reference [22].

The outcome of the fit process is a set of scatter parameters s, $b_{0}$ and l that describe the camera lens as a whole. We thus denote these derived quantities as *integrated scatter parameters*.

#### 2.2.4. Results

In our earlier work, we performed a series of measurements for a selection of eight typical camera lenses with focal lengths ranging from 25 mm to 100 mm [22]. The outcome of that work was an individual set of integrated scatter parameters s, $b_{0}$ and l for each of our camera lenses. We noticed that the scatter parameters for the different camera lenses were of the same order of magnitude and therefore additionally derived a generic set of scatter parameters based on statistical analyses of the individual sets of scatter parameters. This generic set may be applied together with our theoretical model to predict sensor incapacitation by laser light when a camera lens with unknown scatter parameters is used. Note that for this generic set of scatter parameters, we use capital letters:

(19) $$S=-1.86;\;B=0.36{\;\mathrm{sr}}^{-1};\;B_{0}=6.92{\;\mathrm{sr}}^{-1};\;L=2.04\;\mathrm{mrad}$$

## 3. Objective of This Work

After summarizing our earlier work, we come back now to the discussion that we started in the introduction; see Section 1. We mentioned compromises, which we had to introduce to our theoretical model. Here, we will discuss these compromises in detail and then formulate the motivation for this work.

### 3.1. Thoughts Regarding the Theoretical Model

Peterson’s equations (Equations (6) and (7)) allow the use of a distinct set of three scatter parameters s, $b_{0}$ and l, for each individual scattering surface of the camera lens. Furthermore, the size of the light cone $a_{j}$ at each scattering surface is taken into account. In contrast to Peterson’s work, our reduced theoretical scatter model of Equation (8) assumes the same set of scatter parameters for each surface as well as the same beam diameter at each surface. The rationale behind this was, on the one hand, to keep the equation small and practical by getting rid of the summation of Equation (6). One the other hand, we have no knowledge about the size of the light cone at each scattering surface in the case of a COTS camera lens. This would require information about the optical design, which is usually not supplied by the manufacturer. Using our approximation, we can circumvent this lack of knowledge.

Furthermore, Peterson assumes a specific size $a_{j}$ of the light cone in combination with a homogeneous irradiance, which is reasonable if the incident light highly overfills the entrance aperture of the camera lens. This would be the case, e.g., for observation instruments such as astronomical telescopes. In our theoretical considerations for laser safety as well as in our experimental setup, we work with Gaussian laser beams whose beam diameter could be smaller than the entrance pupil of the camera lens. For Equation (8), our theoretical model uses the approach that the whole incident power $P_{\mathrm{in}}$ is distributed within the effective beam diameter $d_{63}$ or the entrance pupil $d_{\mathrm{ap}}$ to calculate the incident irradiance, depending on which diameter is the smaller one [14].

All these assumptions may be seen, of course, as an oversimplification. However, in our earlier work, we showed that the final equation of our model (Equation (4)) is able to describe radial irradiance profiles at the focal plane of camera lenses such as they also occur in reality if the three scatter parameters s, $b_{0}$ and l are known [22]. We would like to emphasize that our theoretical model was designed for the use of laser safety calculations. It is clear that the assumptions and simplifications we make can lead to deviations between theory and reality. In terms of laser safety assessment, this does not pose a problem as long as the laser effects are overestimated, which can be considered a safety factor for this purpose. To ensure this, we took all the respective precautions.

### 3.2. Thoughts Regarding the Experimental Derivation of Scatter Parameters for Camera Lenses

The scatter parameters s, $b_{0}$ (or b) and l in our theoretical model are used to estimate all scatter processes occurring in the camera lens altogether. The complex camera lens is treated as a single scattering element; besides that, we included the factor $N_{\mathrm{ss}}$ to consider the number of scattering surfaces within the camera lens. Since this model is based on the work of Peterson [24], it describes, in principle, only the scattering of light at the smooth surfaces of the optical elements. However, our measurements to derive the scatter parameters (see Section 2.2) also incorporate other effects. These may be other sources of stray light, such as multiple scattering, ghosting effects or scattering from the housing, but also aberrations or diffraction effects resulting from non-circular apertures.

Fortunately, we can ensure that our theoretical model also includes such effects, even if only indirectly, by fitting the model curve to the real data. While it is positive that this works, we have to admit that the integrated scatter parameters we experimentally derived for a camera lens as a whole are not necessarily identical to those that are inherent to the surfaces of its optical elements.

### 3.3. Motivation of This Work

We can now return to the motivation of this work, which we put aside in the introduction. Are the integrated scatter parameters comparable to scatter parameters of single scattering surfaces or not? How reliable is our theoretical model? Will it also work for other camera lenses, apart from the eight lenses for which we derived scatter parameters? All the work presented here was performed to address these questions.

To answer these questions, we decided to compare the output of our theoretical model, fed with experimentally derived scatter parameters, to the output of a stray light analysis software. All software products for stray light simulation, such as FRED or ASAP, support the use of the Harvey scatter model as BSDF for the optical surfaces.

Our initial thought was to take a camera lens with a known optical design, for which we then experimentally determine the integrated scatter parameters as described in Section 2.2. Subsequently, the derived scatter parameters would serve as input for a stray light analysis software to compute the irradiance distribution at the focal plane of the camera lens. If the experimentally derived (integrated) scatter parameters and our underlying theoretical model are reliable, the irradiance distribution simulated by software and the output of our theoretical model should be similar.

Unfortunately, camera lens manufacturers rarely provide information about the optical construction of their lenses. Therefore, we fell back on the two-element air-spaced lens used for laser focusing (LINOS 033486), for which the lens data are contained in the databases of several optical engineering software products. First, we experimentally determined the scatter parameters of this two-element lens. Subsequently, we simulated the irradiance distribution in the lens’ focal plane using the optical engineering software FRED from Photon Engineering, LLC. We then compared the FRED simulation results with the outcome of our theoretical model in order to show that (a) our experimental method for deriving scatter parameters as well as (b) our theoretical model is reliable.

In a second step, we simulated the irradiance distribution at the focal plane of a generic camera lens of the Double-Gauss type using our generic set of scatter parameters; see Equation (19). The design data of the Double-Gauss lens were taken from a standard textbook on optical design [30]. Subsequently, the FRED simulation results were also compared to the outcome of our theoretical model. In this case, the FRED simulation results shall serve to validate that our theoretical model, in conjunction with our generic set of scatter parameters, is able to describe even quite complex multi-element camera lenses as well.

## 4. Experimental Work

**Figure 5:**
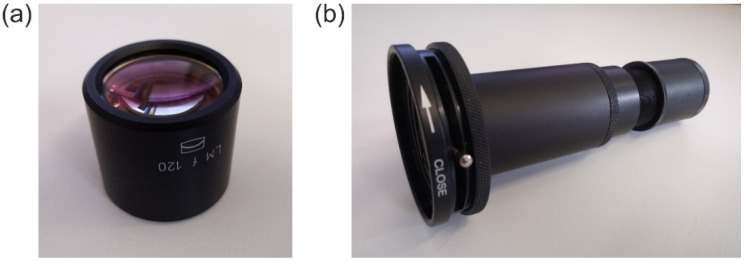
(**a**) Laser focusing optics LINOS 033486. (**b**) Opto-mechanical assembly built using a standard lens tube system for mounting the laser focusing optics to a camera device, with diaphragm at the optics entrance.

In our experimental work, we characterized the focal plane irradiance profile of a two-element laser focusing optics, LINOS 033486. We chose this kind of optics since its optical design was disclosed and therefore allowed us to simulate this optics using the FRED optical engineering software (Section 5). A photograph of this optics is shown in Figure 5a. The optics was integrated into an opto-mechanical assembly consisting of components from a standard lens tube system to attach it to the camera; see Figure 5b.

**Figure 6:**
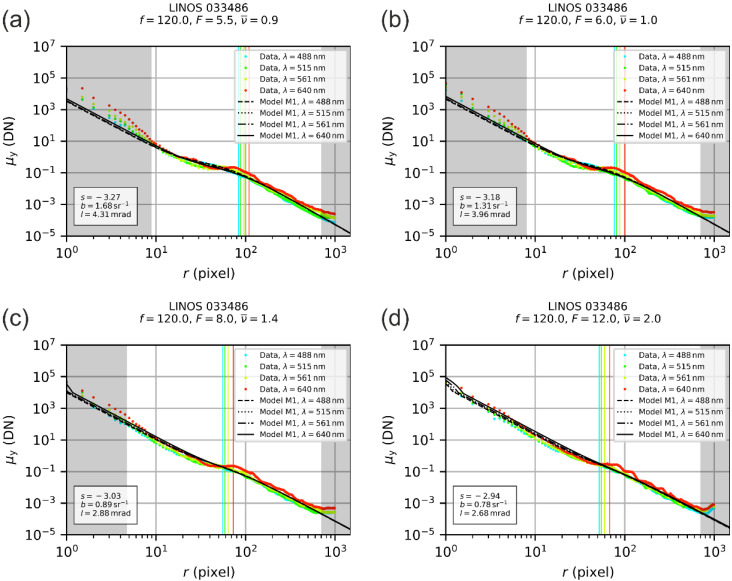
Fit of our theoretical model, according to Section 2.1, to the radial irradiance profiles at the focal plane derived from experimental data for the LINOS 033486 laser focusing lens: (**a**) f-number F=5.5, (**b**) f-number F=6.0, (**c**) f-number F=8.0, (**d**) f-number F=12.0.

The LINOS 033486 laser focusing optics represents an achromat consisting of two air-spaced lenses and has a focal length of 120 mm and a free aperture of 22 mm. Using the anterior iris diaphragm, also shown in Figure 5b, the f-number of the optics could be set. We realized f-numbers of 5.5, 6.0, 8.0 and 12.0 (corresponding to a fully open iris diaphragm and diameters of the iris diaphragm of 21.8 mm, 20 mm, 15 mm and 10 mm). For each f-number, we performed a measurement series and a subsequent fit of our theoretical model as explained in Section 2.2. The results of the fit process are presented in Figure 6, which shows four separate graphs for each specific setting of the f-number F. In each graph, the profile data are plotted as colored data points, where the different colors correspond to the different laser wavelengths of 488 nm, 515 nm, 561 nm and 640 nm. The theoretical models are plotted as black curves. The colored vertical lines mark the value of the scatter parameter l as derived by the fit process.

Some ranges of radial coordinates in Figure 6 are highlighted with a gray background. The data points in these ranges were not included in the fit process. This concerns mainly values of the radial coordinate below 10 pixels distance. For these radial coordinates, we can observe deviations of the measurement results from the theoretical model. Furthermore, we can see from the graphs of Figure 6 that these deviations are stronger for lower f-number values. We attribute these deviations to aberrations of the optics, which are not included in our theoretical model. In order to find out which pixel range should be excluded, the complete fit process comprises a series of three subsequent fits, where the two first fits only serve to find out the exclusion region. The fit process is described in detail in our earlier publication on this topic [22].

For the LINOS 033486 achromat lens, we also excluded radial profile values larger than 700 pixels. From the double-logarithmic plot, we can see that there is an increase in the signal deviating from the expected linear progress of the model curve. Since this deviation would have some impact on the fit result, especially for the scatter parameter s, we also excluded these values for the fit. Currently, we cannot explain the increase in the signal for the larger values of the radial coordinates.

Using the four different fit results, we derived one set of scatter parameters for the LINOS 033486 focusing lens by calculating the median value, which resulted in

(20) $$s=-3.11,\;b=1.1\;{\mathrm{sr}}^{-1},\;l=3.42\cdot 10^{-3}\;\mathrm{rad}.$$

These scatter parameters were applied in our simulation work using the FRED software (rounded to one decimal place); see Section 5.

## 5. Simulation Work

Just as with all software products for stray light simulation, it is necessary to know the exact optical design of the lenses. The basic principle of software for optical engineering is to send a number of rays from a simulated source to the specified optical system and then trace the path of each ray until the rays hit (or miss) the simulated detector (or not). In classical software for optical design, the rays are traced sequentially from the first surface of the optics to the subsequent surfaces, taking into account Snell’s law. From the distribution of the rays at the detector plane, for example, the spot size at the detector plane can be computed.

In the case of stray light analysis using software such as FRED, the beam path is traced using the so-called non-sequential mode. This means that the surfaces of the optical (and mechanical) elements may be hit multiple times by a traced ray in any order until the ray hits the simulated detector array or is removed from the simulation process due to specified exclusion criteria (e.g., maximum allowed number of scatter events per ray). For the simulated light source an output power is specified (in our case: 2 µW), which is distributed among the source rays. When one of the source rays hits an optical or mechanical surface during ray tracing, the ray is split into a number of output rays. This bundle of output rays consists of the regular ray that travels according to Snell’s law (in case an optical element was hit) and a number of scattered rays. The number of created scatter rays as well as the power distribution and the direction in which they are sent depends on the choice of the BSDF for the scattering surface and various other settings of the software.

Finally, all the rays created during the simulation process may hit or miss the simulated detector array. By summing up the power carried by all rays hitting a specific pixel of the simulated detector, the irradiance for this pixel of the detector array will be computed.

Typically, in optics design purposes, a stray light analysis is used to find and reduce the sources of stray light [31]. In that case, the estimation of irradiance values is of minor importance, as it is of major importance to find the origin of stray light. However, in our case, we want to deduce the realistic values of irradiances at the detector, values which can be compared to our experimentally gained results or to the values gained by our theoretical model.

In Section 5.1, we first take a closer look at the optical layout of the two simulated lenses. Then, in Section 5.2, the settings of the FRED software for the simulation are reviewed, especially with regard to the above-mentioned calculation of realistic irradiance values. The way of presenting the simulation results is explained in Section 5.3 by means of an example. The results of the simulation work in its entirety are presented in Section 5.4.

### 5.1. Simulated Lenses

**Figure 7:**
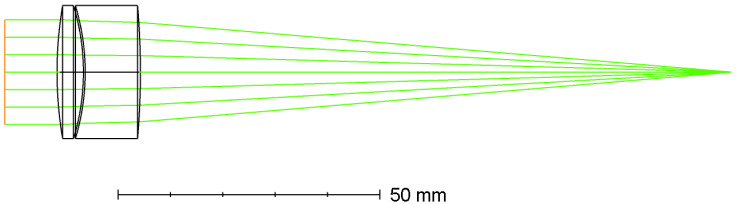
Optical layout of the LINOS 033486 laser focusing lens. Its focal length is 120 mm.

We performed simulations on two very different optics. The first optics was the LINOS 033486 laser focusing lens. This optics represents an achromat and consists of two air-spaced lenses resulting in a focal length of 120 mm. Figure 7 shows the optical layout of this lens system. For the FRED simulation, important lens data are summarized in Table 1.

**Figure 8:**
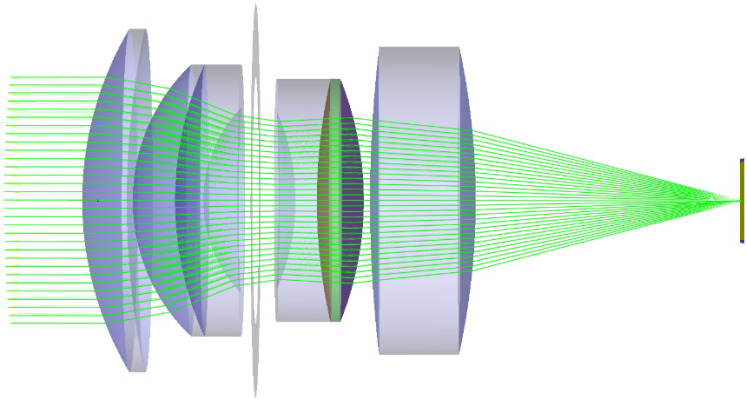
Typical optical layout of a generic Double-Gauss type camera lens.

The second optics simulated is one of the type of the Double-Gauss lens. We chose this lens type because it is a common lens type for consumer SLR cameras (single-lens reflex camera) to which the statement applies: “35-mm SLR normal lenses are invariably Double-Gauss types” [32]. As a representative of such a camera lens, we used the layout published by M. Laikin in his standard textbook on optical design [30]. The optical layout is depicted in Figure 8, and the corresponding design data are summarized in Table 2. The focal length of his camera lens is 35 mm.

### 5.2. Simulation Details

The key element of the simulation is the lens system, whose geometry has to be set up. Accordingly, the lens systems were configured with respect to Table 1 and Table 2.

The next step of the simulation was dedicated to setting up the optical source. For this purpose, FRED offers the possibility to use predefined standard light sources as well as creating detailed customized optical light sources. The use of such a detailed optical source allowed us, on one hand, to match the laser beam used in our experiments as closely as possible and, on the other hand, gave us the possibility of adaptions to different simulation goals, e.g., the number of rays across the source grid. The rays of the source are ordered in a rectangular array of points lying in a plane having an elliptical aperture shape. The total power of the light source was set to 2 µW, and to obtain a Gaussian beam profile, we used a Gaussian apodization.

We chose our light source to be coherent, which means that each ray defined in the source becomes a Gaussian beamlet represented by a base ray and eight secondary rays. FRED performs diffraction and interference calculations using a technique called coherent beam superposition. In the case of coherent beam superposition, any optical fields are modelled by the coherent summation of smaller fundamental beams, which are, in our simulation, generally astigmatic Gaussian beams. Arnaud et al. showed that Gaussian beams can be represented and propagated as real rays, in such a way that real rays can be traced through an optical system while maintaining the representation of the Gaussian beam [33,34]. The near- and far-field diffraction patterns can be computed by coherently summing the rays, represented by Gaussian beamlets traced through the system.

In FRED, analysis planes are used to evaluate the ray distribution on a surface, but they do not interact with the rays during the raytrace. One can choose one or more ray filter criteria in order to select certain types of rays for the analysis. As the default setting for each simulation, we applied an operation, where only rays that hit the detector surface during the raytrace are collected, and those that do not hit the detector surface are discarded. For the case in which we were just interested in the scattered rays, we added an operation that only considered scattered rays that hit the detector surface.

There are different raytrace properties, which we could assign to our geometric objects and define how to propagate every ray that intersects a surface. We generally used the default settings for these raytrace operations. The maximum number of surface intersections for each ray was 1000, and the maximum consecutive intersections that each ray could have with a single surface was 10. The so-called ancestry level cutoff limit was set to 2 for specular rays and to 1 for scatter rays. This setting specifies how many times a given ray is allowed to split due to surface specular or scatter properties.

In reality, a laser beam hitting a lens’ surface would be scattered into the hemisphere (either in transmission or in reflection). To make our simulation more efficient and to obtain a better ray resolution at the analysis surface, i.e., a greater number of rays at each pixel of the detector surface, we defined a direction of interest for the scattered beams. In our case, scattered rays were only generated in a semi angle of 11° of the solid angle cone of the specular direction of the source ray. This method is called importance sampling.

After passing the optical lens system, the light rays have to be analyzed. Therefore, we created a detector element by using a plane element with a semi-aperture size of 2.75 mm and assigned a so-called analysis surface with a size of 5 mm divided in 1000 divisions to the plane element. With these parameters, the detector we used experimentally (Allied Vision Mako G-419B NIR utilizing a CMOSIS/ams CMV4000 NIR imaging sensor, with a pixel size of 5.5 µm) was best described.

#### 5.2.1. Simulation Settings

The simulated signal at the detector array is composed of two contributions: (1) the contribution from the regular rays that are not scattered and (2) the contribution of the scattered rays. Let us assume that we would perform a simulation with only a single source ray, to which we assign the complete power of 2 µW. When we perform the simulation without considering light scattering, this ray would hit one of the pixels of the simulated detector. Thus, the simulated detector image would show no signal with the exception of the one pixel that was hit. This means that we need an adequate number of source rays in order to simulate the signal arising from the regular rays. These regular rays will determine the irradiance distribution of the focused laser spot, which then can be compared to the diffraction component $E_{d}$ of our theoretical model.

The same applies to the scattered rays. If we want to simulate a realistic detector signal produced by stray light, we need a large number of scatter rays. The scatter rays will determine the irradiance distribution far away from the center of the laser spot. The number of scatter arrays arriving at the simulated detector array should be far larger than the number of detector pixels.

Unfortunately, a large number of source rays combined with a large number of generated scatter rays places extreme demands on the computation time as well as computer memory. Using a standard computer, a simulation with such optimal settings would take up too much time. Therefore, some restrictions had to be made to the simulation settings in order to perform the simulations in an adequate time (<several weeks).

In the course of our work, we derived three sets of parameters for the FRED software:

- Set 1: A set of parameters designed to describe the irradiance distribution originating from the regular rays (diffraction) as well as the scattered rays (stray light) with reasonably accuracy.
- Set 2: A set of parameters dedicated to simulating properly the irradiance component due to scattered rays (stray light).
- Set 3: A set of parameters dedicated to simulating properly the irradiance component that origins from the regular rays (diffraction).

The values for these three settings for the FRED software are listed in Table 3. For the LINOS 033486 laser focusing lens, we performed simulations for three values of the f-number: F=6.0, F=8.0 and F=12.0. As scatter parameters, we used the values gained from our experimental work; see Section 4.

For the 35 mm Double-Gauss lens, simulations were performed for four f-numbers: F=2.8, F=5.6, F=8.0 and F=16.0. As scatter parameters, we used our generic set of scatter parameters estimated in earlier work (see Section 2.2.4), as we do not have such a lens at our disposal for which we know the exact technical specifications.

#### 5.2.2. Simulation Output

The output of a FRED simulation is the irradiance values that are present at each pixel of the simulated detector array. Typically, these irradiance values are represented as an image showing the spatial distribution of the irradiance. Since the range of irradiance values often spans several orders of magnitude, such images usually show the logarithmized values of the irradiance. In this publication, we always show irradiance values related to a value of 1 W/mm² in unit of decibels: $10\cdot \log_{10}\left(\frac{E}{1\;W/{\mathrm{mm}}^{2}}\right)$ for the spatial irradiance distributions we generated.

Since our theoretical model only predicts radially symmetrical irradiance distributions, we additionally present the simulation results as a scatter plot, where the irradiance of each detector pixel is plotted versus its radial distance (in pixels) to the center of the simulated laser spot.

### 5.3. Example of the Simulation Results

In this section, we present in detail examples of our simulation results based on the different settings for a specific setup, namely the LINOS 033486 lens for an f-number of F=12.

#### 5.3.1. Example 1: Simulation Settings—Set 1

**Figure 9:**
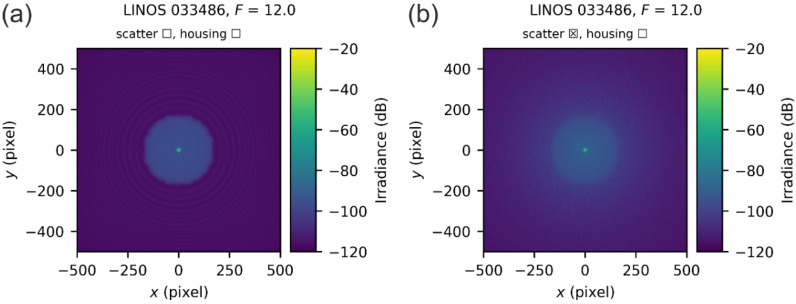
Simulated irradiance distribution at the focal plane of the LINOS 033486 lens for an f-number of F=12: (**a**) simulation neglecting light scattering and (**b**) simulation, which takes light scattering at the surfaces of the optical elements into account. As simulation parameters, set 1 of the simulation settings was applied.

In Figure 9, two examples of simulated spatial irradiance distribution for the LINOS 033486 lens are shown using set 1 of the simulation settings according to Table 3. For the result presented by Figure 9a, light scattering at the surfaces of the optical elements was neglected, whereas it was considered in the case of Figure 9b. In the further course of this publication, the check boxes contained in the figure titles always indicate to the reader whether the simulation was considered light scattering and whether a housing was included in the layout or not. In the examples of Figure 9, a housing was not considered, which means that only scatter at the optical elements was taken into account.

**Figure 10:**
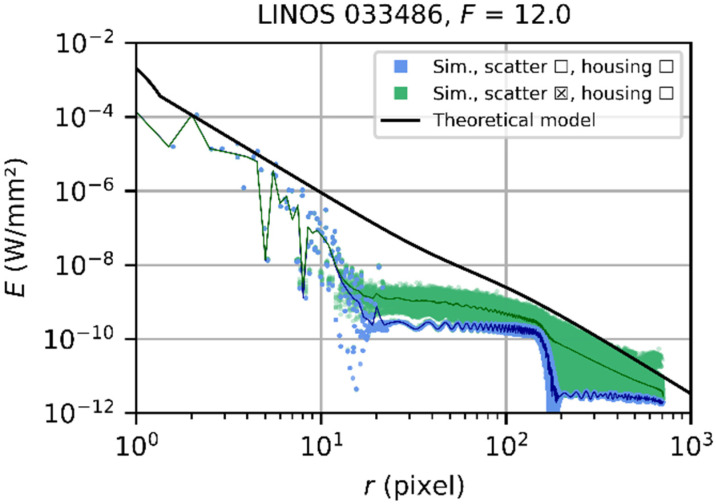
Scatter plot of simulated irradiance values for all detector pixels versus their radial distance to the center of the simulated laser spot for the LINOS 033486 lens and an f-number F=12. Set 1 of simulation settings was applied.

For the examples of Figure 9, the corresponding scatter plots of the radial irradiance distributions are shown in Figure 10. Each colored data point represents one detector pixel. The two cases of the example above are represented by different colors: in blue, the case where light scattering was neglected (corresponding to Figure 9a) and in green, the case with light scattering (corresponding to Figure 9b). We can see that the data points do not appear as a simple curve but as colored bands since the different detector pixels are hit by a different number of simulated rays. In addition to the colored data points, for both cases, a solid curve of similar color (but darker) is plotted into the graph, which depicts the mean value of the colored band. Finally, we also plot the radial irradiance profile as predicted by our theoretical model as a thick black solid curve.

We can see in the example of Figure 10 that the theoretical model curve manifests as the envelope of the simulation results for small values of the radial coordinate (r<10 px.) and for large values (r>200 px.). There is a deviation for radial coordinates roughly between 10 and 200 pixels, which we can explain by the low number of source rays (973, see Table 3). As we will see in Section 5.3.3, the use of an adequate number of source rays results in a good agreement of the theoretical model and simulation.

#### 5.3.2. Example 2: Simulation Settings—Set 2

**Figure 11:**
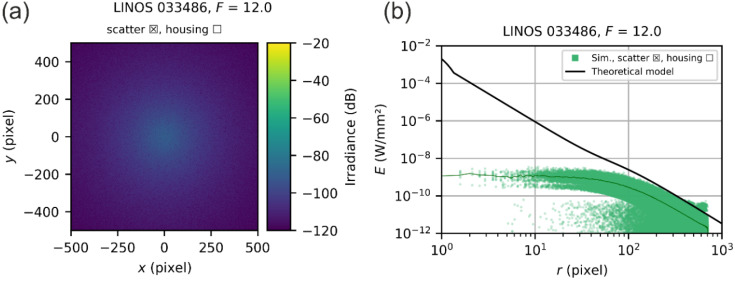
Simulation results for the LINOS 033486 lens and an f-number F=12 using the scatter-focused parameter set 2 with a low number of source rays but a high number of scattered rays. Only the scatter rays were analyzed. (**a**) Results represented as an image. (**b**) Results shown as a scatter plot. Please note: The curve of the theoretical model estimates the signal of both the diffractive component (regular rays) and the scatter component.

In this example, we simulated the focal plane irradiance of the LINOS 033486 laser focusing lens using the scatter-focused parameter set 2 with a low number of source rays (973) but a quite large number of scattered rays per scatter event (250,000). In Figure 11, the simulated spatial irradiance distribution (Figure 11a) and the corresponding scatter plot (Figure 11b) are shown. For this result, only the scatter rays arriving at the detector were analyzed, while the regular rays were neglected for this simulation.

The irradiance distribution for low values of the radial coordinate r is not properly simulated since the central detector pixels are not hit by enough rays. This is the region where the diffraction components dominate the signal. However, we can see in the scatter plot that the black solid curve representing the theoretical model fits quite well to the irradiance values for larger values of the radial coordinate r (>100 pixel). For these radial coordinates, the number of rays hitting the detector pixels seems to be large enough, some of them matching the predicted irradiance by the theoretical model. The theoretical model represents the envelope of the simulated irradiance in this region. This means that the scatter component of the irradiance distribution can be simulated quite well using a simulation configuration with a high number of scatter rays.

#### 5.3.3. Example 3: Simulation Settings—Set 3

**Figure 12:**
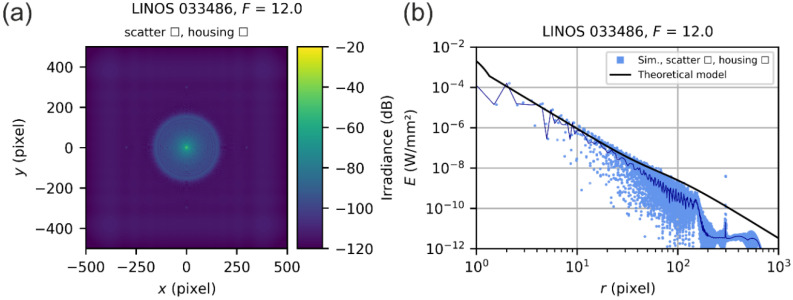
Simulation results for the LINOS 033486 lens setting an f-number of F=12 and using the diffraction-focused parameter set 3 with a high number of source rays. Only the regular rays were analyzed, scattered rays were neglected. (**a**) Results represented as an image. (**b**) Results shown as a scatter plot.

In Figure 12, the results of a simulation of the same system as before is shown but using diffraction-focused parameter set 3 with a large number of source rays (196,364). For this investigation, only those regular rays arriving at the detector were analyzed. The scatter rays were neglected for the diffraction-focused simulation.

We can see now that the simulated signals for small values of the radial coordinate (<100 pixel) match quite well with the theoretical calculation. This means that the diffraction component can be simulated quite well by using a simulation configuration with a large number of source rays.

#### 5.3.4. Multi-Step Simulations

The computer system we used for the simulations did not allow to simulate the entire detector signal adequately with a single simulation run using a single set of simulation settings. The best set of parameters we found is listed in Table 3 as set 1, and a corresponding example was presented in Section 5.3.1.

**Figure 13:**
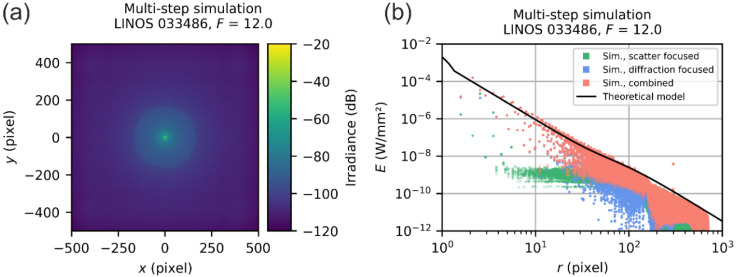
Simulation results for the combination of two simulation results for the LINOS 033486 lens when setting an f-number of F=12: Green data points: simulation using the scatter-focused parameter set 2, blue data points: simulation using the diffraction-focused parameter set 3, red data points: combined simulation results. (**a**) Results represented as an image. (**b**) Results shown as a scatter plot.

Quite good simulation results were achieved by combining the two simulation results for the scatter-focused set 2 and the diffraction-focused set 3. As an example, the results achieved in Section 5.3.2 and Section 5.3.3 and the combination of both are presented in Figure 13. For the combination, the diffraction-focused data were scaled by the fraction of diffracted power $\eta_{d}$ as given by Equation (17) and then added to the scatter-focused data. The result is presented in Figure 13 by the red data points. We can see that the theoretical curve predicts quite well the envelope of the combined simulation results.

In the further course, we denote this kind of simulation as *multi-step simulation* since it comprises more than one simulation run to create the result.

### 5.4. Overview of All Simulation Results

As already pointed out above, raytrace simulations require a lot of computing time, even if the parameter sets used are considered optimal. Another aspect concerns the simulation results obtained with it. As we experienced in Section 5.3.1 when using the set 1 of parameters, deviations occur between the simulation results and the prediction of the theoretical model, especially for radial values between ~10 and 100 pixels. This deviation is due to the inadequate number of sources rays of set 1 of simulation settings. Using an adequate number of sources rays (e.g., 196,364 as in the case of the diffraction-focused parameter set 3 of simulation settings) would lead to calculation times of several weeks, as long as we use the computers just available to us and keep a high number of scatter rays. The way out is combining two specific simulation runs: one for the diffraction component and one for the scatter component. This combination proved to be much more effective.

Thus, in the following, we would like to focus on our results concerning the LINOS 033486 laser focusing optics (Section 5.4.1) and the generic 35 mm Double-Gauss lens (Section 5.4.2) gained with the multi-step simulation. Please note that we usually neglected the housing of the camera lens in our stray light simulations. The reason for this was that we could not see any difference between simulation results with lens housing and without lens housing. More details on this topic can be found in Appendix A.

#### 5.4.1. Simulation Results for the Two-Element Laser Focusing Optics

**Figure 14:**
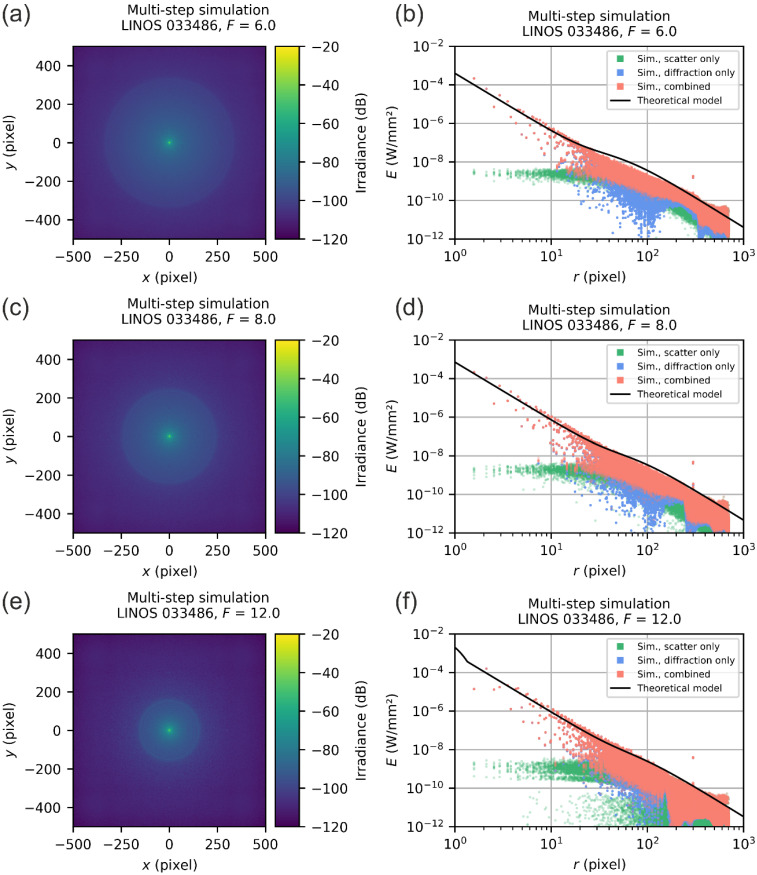
Results of the multi-step simulation for the LINOS 033486 laser focusing optics for the f-numbers of F=6.0, F=8.0 and F=12.0. (**a**,**c**,**e**) Results represented as an image. (**b**,**d**,**f**) Results shown as a scatter plot.

The multi-step simulations for the LINOS 033486 laser focusing optics were performed for three values of f-numbers: F=6.0, F=8.0, and F=12.0. The results are presented in Figure 14. On the left-hand side, the simulated spatial irradiance distributions are shown and on the right-hand side, the corresponding scatter plots of the radial irradiance distributions. For the scatter plots, we used different colors to indicate the different simulation results: blue data points represent the result of the diffraction-focused simulations, whereas the green data points correspond to the scatter-focused simulations. The combined results of the multi-step simulation are shown by red data points.

The theoretical predictions match remarkably well the envelope of the simulation data for all three simulated f-numbers. Of course, this is not surprising for this focusing lens since it has a rather simple optical design. The beam diameters do not vary that much for the different scattering surfaces; thus, the simplifications of our theoretical model (e.g., equal beam diameter at every scattering surface) have little negative effects. However, at this point, we can conclude that our simple theoretical model is able to predict stray light irradiance accurately for simple optical systems.

#### 5.4.2. Results for a Generic 35 mm Double-Gauss Lens

**Figure 15:**
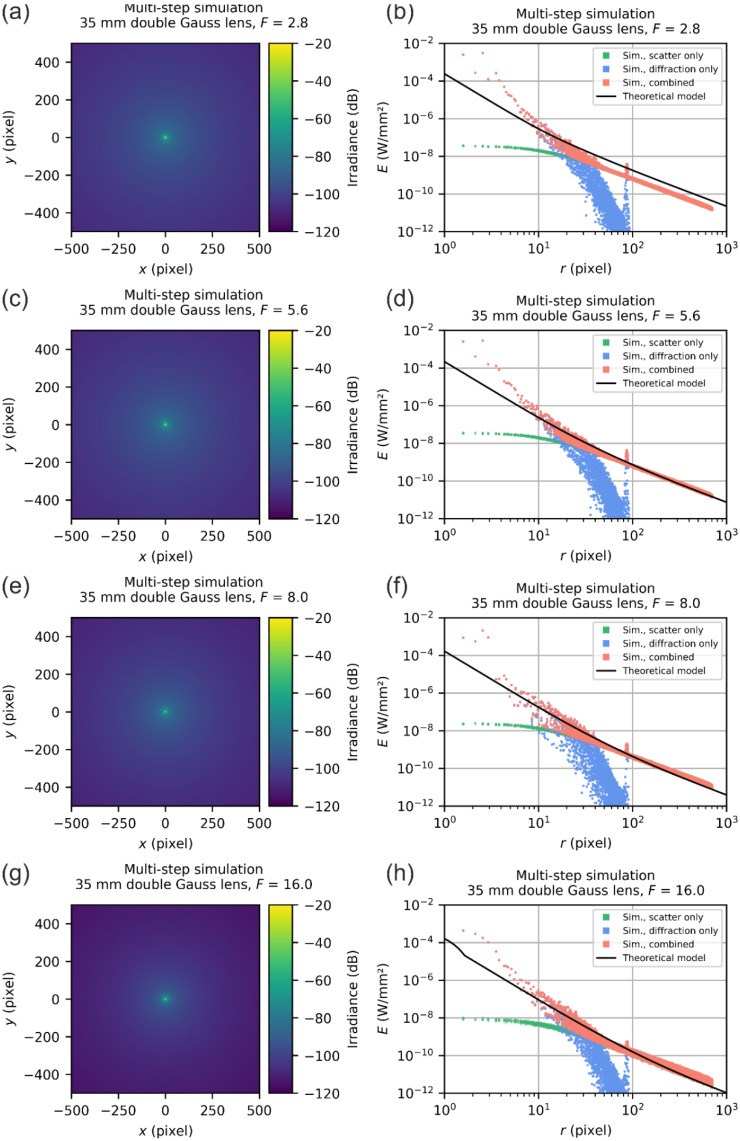
Results of the multi-step simulation for a 35 mm Double-Gauss lens for the f-numbers of F=2.8, F=5.6, F=8.0 and F=16.0, using our generic set of scatter parameters. (**a**,**c**,**e**,**g**) Results represented as an image. (**b**,**d**,**f**,**h**) Results shown as a scatter plot.

Since we had no lens of the Double-Gauss type at hand, for which we know the exact technical specifications, we switched to a generic Double-Gauss lens with a focal length of 35 mm, as already stated above. At the same time, this meant that we had no dedicated scatter parameters that we could use. However, we could fall back on a generic data set of scatter parameters that is available to us from our earlier measurements on lenses of the Double-Gauss type: it is the generic set of integrated scatter parameters as listed by Equation (19). Furthermore, the stray light irradiance simulations were performed for four f-numbers: F=2.8, F=5.6, F=8.0 and F=16.0. The simulation results are presented in Figure 15.

As one may recognize, for radial coordinates < 10 pixels, there is a larger deviation between the results of the model and the simulations, which we attribute to aberrations that are not covered by our theoretical model.

However, for very small dazzle spots, which cover only a few pixels, we can tolerate such errors as long as the accuracy is given for large dazzle spots.

For radial coordinates larger than ~10 pixels, we can see from the scatter plots that the theoretical model fits reasonably to the simulation results. For small f-numbers, see Figure 15b, there seems to be an overestimation of the irradiance while for large f-numbers, see Figure 15h, an underestimation occurs. An overestimation is acceptable in terms of laser safety and can be seen as a safety factor, whereas an underestimation may pose a problem. Fortunately, for the intended purpose of our model—the quantitative description of sensor incapacitation by laser radiation—the model output is reasonable.

Note that in the scatter plots of Figure 15, we can see a peak in the irradiance values for the diffraction-focused simulation at a radial coordinate of ~90 pixels. A similar peak occurs in the simulation for the LINOS 033486 laser focusing optics at radial coordinate of ~300 pixels (see Figure 14), which is only noticeable as a red outlier. Currently, we have no explanation for the appearance of this peak. Fortunately, the peak has little influence on the results of the multi-step simulation results since the diffraction irradiance is orders of magnitudes lower than the scatter irradiance for these radial coordinates. We assume that the peaks are a simulation artefact without a real physical background.

## 6. Summary

The objective of the presented work was to validate our simplified theoretical model for the estimation of the irradiance distribution at the focal plane of commercial off-the-shelf (COTS) camera lenses. This theoretical model was explicitly developed for laser safety calculations for imaging sensors. It was developed such that you only need to perform relatively simple calculations based on closed-form equations in order to estimate the diffraction and scatter characteristics of light of camera lenses. When deriving the theoretical model, emphasis was placed on simplicity rather than accuracy in order to enable unexperienced users to work with the derived equations. This required several simplifications, which led to the question of how accurate the model actually is. To answer this question, we compared the output of our theoretical model to stray light simulations using the popular optical design software FRED. The simulations were performed for two different optics, a well-known two-element laser focusing lens and a generic lens of the Double-Gauss type.

In the case of the two-element laser focusing lens, we measured its scatter parameters beforehand and fed these scatter parameters into both the theoretical model and the simulation software. For this type of lens, we found a very high agreement between the two results.

For the Double-Gauss lens, we used a generic set of scatter parameters, derived in our earlier work. This generic set of scatter parameters can be applied if no appropriate numerical values are available. Also in this case, we found an adequate agreement between our simplified theoretical model and the output of corresponding stray light simulation. We found an overestimation of the focal plane irradiance rather than an underestimation, which is good in the sense of laser safety. We attribute the differences between the theoretical model and the simulations to the simplifications applied in deriving the theoretical model. Despite the simplifications we made to derive our theoretical model (e.g., a constant size of the light cones within the optics), we can state that the model provides reasonable results in the context of the issues we are considering.

Further work will focus on the experimental validation of our theoretical model, in particular on the applicability of the generic set of scatter parameters. Nevertheless, we also want to improve the framework for laser safety calculations. In particular, the calculation of hazard distances, based on our theoretical model.

## Figures and Tables

**Table 1:** Data of the LINOS 033486 laser focusing lens.

Surface	Radius (mm)	Thickness (mm)	Material	Diameter (mm)
1	Stop	10.0		var.
2	75.531	5.038	S-NSL5	12.7
3	−43.714	0.348		12.7
4	−43.714	10.544	S_TIM22	12.7
5	−147.490	112.864		12.7

**Table 2:** Data of the generic Double-Gauss type camera lens we used. Its focal length is 35 mm.

Surface	Radius (mm)	Thickness (mm)	Material	Diameter (mm)
1	23.82	3.20	N-LAK33	24.38
2	58.50	0.38		24.38
3	13.31	3.01	N-LAK33	19.30
4	23.46	1.99	SF1	19.30
5	9.43	3.72		13.21
6	Stop	2.82		9.35
7	−15.76	1.52	F5	12.70
8	38.67	3.33	N-LAK33	17.27
9	−23.21	0.44		17.27
10	92.34	7.51	N-LAK33	21.84
11	−51.06	19.05		21.84

**Table 3:** Parameter sets for the FRED software to simulate the irradiance distribution at the focal plane of camera lenses.

Parameter	Simulation Parameter Set		
	Set 1: Diffraction + Scatter	Set 2: Scatter-Focused	Set 3: Diffraction-Focused
**Source**			
wavelength	550 nm		
beam shape	Gaussian		
beam diameter $d_{86}$	20 mm		
power	2 µW		
coherence	yes		
polarization	no		
incidence angle	0°		
semi-aperture of the source grid	10 mm		
number of source rays	973	973	196,364
**LINOS 033486 lens**			
focal length f	120 mm		
f-numbers F	6/8/12		
scatter parameters	$s=-3.11,\;b=1.1\;{\mathrm{sr}}^{-1},\;l=3.42\;\mathrm{mrad}$		
**35 mm Double-Gauss lens**			
focal length f	35 mm		
f-numbers F	2.8/5.6/8/16		
scatter parameters	$S=-1.86,\;B=0.36{\;\mathrm{sr}}^{-1},\;L=2.04\;\mathrm{mrad}$		
**Scatter model**			
lens surfaces	Harvey-Shack		
lens edges/mechanical parts	Black Lambertian (4% reflectivity)		
number of scatter rays	250,000	250,000	0
**Importance sampling**			
lens surfaces	Solid cone with 11° semi-angle		
lens edges/mechanical parts	FRED default		
**Detector**			
pixels	1000 × 1000		
pixel size	5.5 µm		
considered rays	regular + scattered	scattered only	regular only

**Figure A1:**
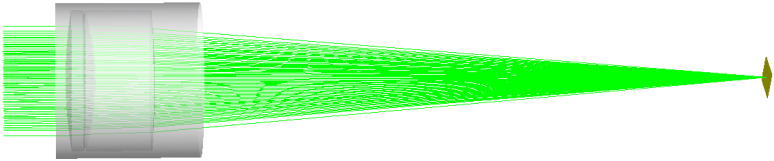
Optical layout of the LINOS 033486 laser focusing lens with a housing.

**Figure A2:**
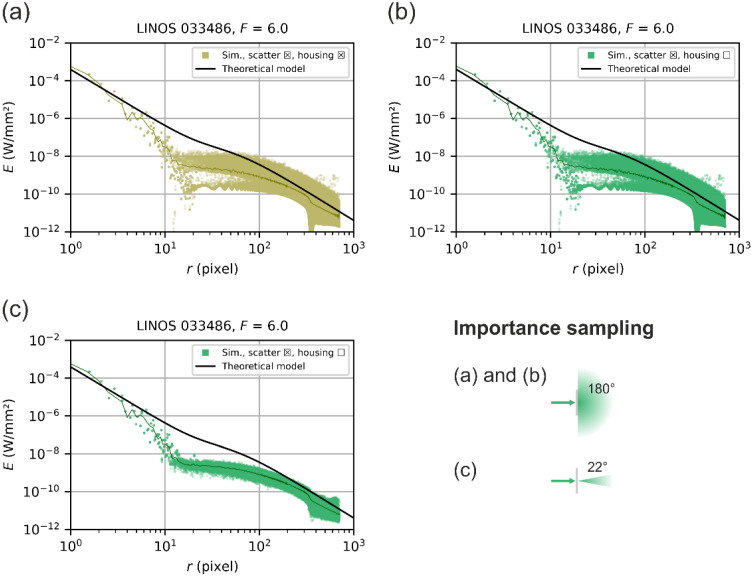
Simulation result for the LINOS 033486 lens at an f-number of F=6. (**a**) Simulation considering housing and using adapted simulation settings. (**b**) Simulation without housing and using adapted simulation settings. (**c**) Simulation without housing using set 1 of simulation settings. The inset on the bottom right shows which importance sampling setting was used for each simulation.

## Appendix A. Stray Light from the Lens Housing

In our stray light simulations, we usually neglected the housing of the camera lens. The reason for this was that we could not see any difference between simulation results with lens housing and without lens housing. There are several reasons that can explain this fact.

In our theoretical model, the laser light is supposed to incite along the optical axis of the camera lens and that the focal spot hits the imaging sensor centrally. This was also the basis for our simulation work. However, this implies that no regular ray will hit the housing of the camera lens; see Figure 7 and Figure 8. Thus, stray light originating from the housing can only be caused by a second-order scatter event. Typically, the power contained within a second-order scatter rays is so low that FRED standardly dismisses these rays. Furthermore, importance sampling is used in stray light simulation, which means that only scatter rays are traced that travel in a specified direction. In our case, importance sampling includes only scatter rays that travel within a cone with a semi-angle of 11°, which means that only few scatter rays will ever hit the housing. We can conclude that for our simulations, the effects of the housing are negligible. Nevertheless, we performed dedicated simulation runs to support this assumption.

We investigated were carried out using the LINOS 033486 laser focusing optics as an example. For this lens, a housing was implemented in the FRED software; see Figure A1. Comparative simulations including the housing and without it were performed. For these simulations, we used, in principle, set 1 of the simulation settings. However, regarding the importance sampling, we allowed here the scattering of rays in the full half space (related to the specular direction of the regular ray). Thus, scattered rays definitely hit the housing.

Scatter plots of the simulation results are shown in Figure A2: on the left-hand side (Figure A2a), the results considering the housing; and on the right-hand side (Figure A2b) the results, neglecting the housing. We can clearly see that there is no difference visible. In Figure A2c, we show for comparison additionally the results using the set 1 of simulation settings from Table 3.

As a side note, we can observe that the distribution of the data points of Figure A2a,b using the adapted settings differs noticeably from the distribution of Figure A2c, which is the result using the set 1 of simulation settings.

## References

[1] Lewis G.D., Chretien S., Santos C.N., Vandewal M., Hackens B. (2018). In-band low-power laser dazzle and pixel damage of an uncooled LWIR thermal imager. Proc. SPIE.

[2] Santos C.N., Chrétien S., Merella L., Vandewal M. (2018). Visible and near-infrared laser dazzling of CCD and CMOS cameras. Proc. SPIE.

[3] Özbilgin T., Yeniay A. (2018). Laser dazzling analysis of camera sensors. Proc. SPIE.

[4] Lewis G.D., Santos C.N., Vandewal M. (2019). Mitigation of laser dazzle effects on a mid-wave infrared thermal imager by reducing the integration time of the focal plane array. Proc. SPIE.

[5] Steinvall O. (2021). The potential role of laser in combating UAVs: Part 2; laser as a countermeasure and weapon. Proc. SPIE.

[6] Toet A., Benoist K.W., van Lingen J.N.J., Schleijpen H.R.M.A. (2013). Optical countermeasures against CLOS weapon systems. Proc. SPIE.

[7] Titterton D.H. (2010). Application of laser technology to optical countermeasures. Imaging Sci. J..

[8] Titterton D.H. (2004). A review of the development of optical countermeasures. Proc. SPIE.

[9] (2015). ANSI Z136.6-2015.

[10] (2018). TROS Laserstrahlung Teil 2: Messungen und Berechnungen von Expositionen gegenüber Laserstrahlung, July 2018.

[11] Williamson C.A., McLin L.N. (2015). Nominal ocular dazzle distance (NODD). Appl. Opt..

[12] Williamson C.A., McLin L.N. (2018). Determination of a laser eye dazzle safety framework. J. Laser Appl..

[13] Williamson C.A., McLin L.N. (2017). Laser eye dazzle safety framework. Proceedings of the International Laser Safety Conference.

[14] Ritt G. (2019). Laser Safety Calculations for Imaging Sensors. Sensors.

[15] Schwarz B., Ritt G., Koerber M., Eberle B. (2017). Laser-induced damage threshold of camera sensors and micro-optoelectromechanical systems. Opt. Eng..

[16] Schwarz B., Koerber M., Ritt G., Eberle B. (2019). Further investigation on laser-induced damage thresholds of camera sensors and micro-optomechanical systems. Proc. SPIE.

[17] Schwarz B., Ritt G., Eberle B. (2021). Impact of threshold assessment methods in laser-induced damage measurements using the examples of CCD, CMOS, and DMD. Appl. Opt..

[18] Théberge F., Auclair M., Daigle J.-F., Pudo D. (2022). Damage thresholds of silicon-based cameras for in-band and out-of-band laser expositions. Appl. Opt..

[19] Westgate C., James D. (2022). Visible-Band Nanosecond Pulsed Laser Damage Thresholds of Silicon 2D Imaging Arrays. Sensors.

[20] Schleijpen R.M.A., van den Heuvel J.C., Mieremet A.L., Mellier B., van Putten F.J.M. (2007). Laser dazzling of focal plane array cameras. Proc. SPIE.

[21] Benoist K.W., Schleijpen R.M.A. (2014). Modelling of the over-exposed pixel area of CCD cameras caused by laser dazzling. Proc. SPIE.

[22] Ritt G., Schwarz B., Eberle B. (2020). Estimation of Lens Stray Light with Regard to the Incapacitation of Imaging Sensors. Sensor.

[23] FRED Software Photon Engineering. https://photonengr.com/fred-software/.

[24] Peterson G.L. (2004). Analytic expressions for in-field scattered light distributions. Proc. SPIE.

[25] Fest E.C. (2013). Stray Light Analysis and Control.

[26] Pfisterer R.N. (2011). Approximated Scatter Models for Stray Light Analysis. Opt. Photonics News.

[27] Scattering in ASAP. http://www.breault.com/knowledge-base/scattering-asap.

[28] Urey H. (2004). Spot size, depth-of-focus, and diffraction ring intensity formulas for truncated Gaussian beams. Appl. Opt..

[29] EMVA Standard 1288, Standard for Characterization of Image Sensors and Cameras, Release 3.1. European Machine Vision Association. 30. December 2016. https://www.emva.org/standards-technology/emva-1288/emva-standard-1288-downloads.

[30] Laikin M. (2007). Lens Design.

[31] Pompea S.M., Pfisterer R.N., Ellis S., Arion D.N., Fienberg R.T., Smith T.C. (2010). Optical and system engineering in the development of a high-quality student telescope kit. Proc. SPIE.

[32] Betensky E., Kreitzer M., Moskovich J., Bass M. (1995). Camera lenses. Handbook of Optics, Volume II, Devices, Measurements, and Properties.

[33] Arnaud J. (1985). Representation of Gaussian Beams by Complex Rays. Appl. Opt..

[34] Arnaud J., Kogelnik H. (1969). Gaussian Light Beams with General Astigmatism. Appl. Opt..

